# Detection of donor-derived cell-free DNA in the setting of multiple kidney transplantations

**DOI:** 10.3389/fimmu.2024.1282521

**Published:** 2024-02-22

**Authors:** Linnea Pettersson, Lukas Frischknecht, Sofia Westerling, Hamid Ramezanali, Lukas Weidmann, Kai Castrezana Lopez, Thomas Schachtner, Jakob Nilsson

**Affiliations:** ^1^ Devyser AB, Stockholm, Sweden; ^2^ Department of Immunology, University Hospital Zurich (USZ), Zurich, Switzerland; ^3^ Division of Nephrology, University Hospital Zurich (USZ), Zurich, Switzerland

**Keywords:** kidney transplantation, donor derived cell free DNA, Dd-cfDNA, ABMR, graft loss, second kidney transplantation, NGS

## Abstract

**Background:**

The routine use of donor-derived cell-free DNA (dd-cfDNA) assays to monitor graft damage in patients after kidney transplantation is being implemented in many transplant centers worldwide. The interpretation of the results can be complicated in the setting of multiple sequential kidney transplantations where accurate donor assignment of the detected dd-cfDNA can be methodologically challenging.

**Methods:**

We investigated the ability of a new next-generation sequencing (NGS)-based dd-cfDNA assay to accurately identify the source of the detected dd-cfDNA in artificially generated samples as well as clinical samples from 31 patients who had undergone two sequential kidney transplantations.

**Results:**

The assay showed a high accuracy in quantifying and correctly assigning dd-cfDNA in our artificially generated chimeric sample experiments over a clinically meaningful quantitative range. In our clinical samples, we were able to detect dd-cfDNA from the first transplanted (nonfunctioning) graft in 20% of the analyzed patients. The amount of dd-cfDNA detected from the first graft was consistently in the range of 0.1%–0.6% and showed a fluctuation over time in patients where we analyzed sequential samples.

**Conclusion:**

This is the first report on the use of a dd-cfDNA assay to detect dd-cfDNA from multiple kidney transplants. Our data show that a clinically relevant fraction of the transplanted patients have detectable dd-cfDNA from the first donor graft and that the amount of detected dd-cfDNA is in a range where it could influence clinical decision-making.

## Introduction

Kidney transplantation is a life-saving treatment for individuals with end-stage renal disease (ESRD). In recent years, significant advancements have been made in the field of transplant medicine, improving the success rates and long-term outcomes of kidney transplants (1). Among these breakthroughs, the utilization of donor-derived cell-free DNA (dd-cfDNA) has emerged as a promising tool to improve the monitoring and management of kidney transplant patients post-transplantation (2–4). dd-cfDNA refers to the fragments of genetic material released from the transplanted organ, which circulate freely in the recipient’s bloodstream. These fragments are a result of cellular turnover and organ injury during the transplantation process. By analyzing and quantifying the levels of dd-cfDNA, clinicians can gain valuable insights into the health and function of the transplanted graft (5). A significant advantage of dd-cfDNA lies in its noninvasive nature, offering a means of monitoring and diagnosing kidney allograft rejection without the need for invasive biopsies. Traditional biopsy procedures can be uncomfortable for patients and carry risks while providing only a limited representation of overall graft health (6). The analysis of dd-cfDNA levels can be used to monitor overall graft health where low or undetectable levels would indicate that there is no current ongoing graft injury (4). In cases of elevated dd-cfDNA, the pattern and kinetics of the elevation can also be suggestive of the type of graft injury that is occurring. As an example, antibody-mediated rejection (ABMR) associated with the detection of donor-specific anti-HLA antibodies (DSA) typically leads to a prolonged and significant elevation of dd-cfDNA values, whereas T cell-mediated rejection (TCMR) has a more variable association with dd-cfDNA values which is dependent on the molecular profile of the TCMR (7). The detection of pre-transplant or *de novo* DSA has been consistently shown to be associated with worse prognosis in the setting of both deceased and living donor kidney transplantations (8–10). The development of *de novo* DSA is also widely used currently in post-transplant monitoring, but a recent study suggested that dd-cfDNA has a higher predictive value for biopsy-proven ABMR, likely due to the existence of a relatively large population of DSA-negative ABMR cases (11). Recent data have also shown that a detected elevation in dd-cfDNA precedes the detection of *de novo* DSA, which suggests that graft damage is already ongoing at the time of DSA detection (12). Even if the likely underlying pathology cannot be inferred from the pattern of dd-cfDNA elevation, it can still identify individuals at a higher risk of graft loss, which allow clinicians to adjust their post-transplant management plans accordingly, leading to a more individualized approach and improving the selection of individuals for more invasive interventions such as graft biopsies (5). However, despite the significant advancements in utilizing dd-cfDNA in kidney transplantation, methodological challenges can arise in the context of a second kidney transplantation, where it can be challenging to determine from which allograft the potentially elevated dd-cfDNA originated. The ability to differentiate between dd-cfDNA from the first and second transplants therefore becomes crucial for accurate monitoring and diagnosis in these individuals. The number of individuals who are receiving a second kidney transplant has been increasing in the last 20 years (13). At our institution, between 2000 and 2017, approximately 15% of all kidney transplantations were performed on individuals who had received at least one prior kidney (14). In a majority of these individuals, the first graft will not be explanted and could therefore be a source of dd-cfDNA. It is also known that even after explanting a previously transplanted failing kidney, there is a continued risk of alloimmunization, likely due to donor tissue that is still present in the recipient after explantation (15). This is the reason why immunosuppression is usually continued in such individuals, to prevent the formation of anti-HLA antibodies that could make it more difficult to find a suitable donor for a second kidney transplantation (16). The ability to accurately assign detected dd-cfDNA sequences in the setting of multiple donor grafts relies on the method used, where the number of informative markers plays a crucial role. The genetic markers in chimerism assays are selected based on their frequency in the general population, and with an increasing number of grafts from different donors, there is a need for more markers that may be informative in such a setting (17). We present data on a new next-generation sequencing (NGS)-based method for dd-cfDNA analysis wherein the use of 50 indel markers can accurately detect dd-cfDNA from separate grafts in both experimentally generated samples containing cfDNA from several individuals and actual clinical samples from individuals with a second kidney transplant.

## Methods

### Study samples

**Figure 1:**
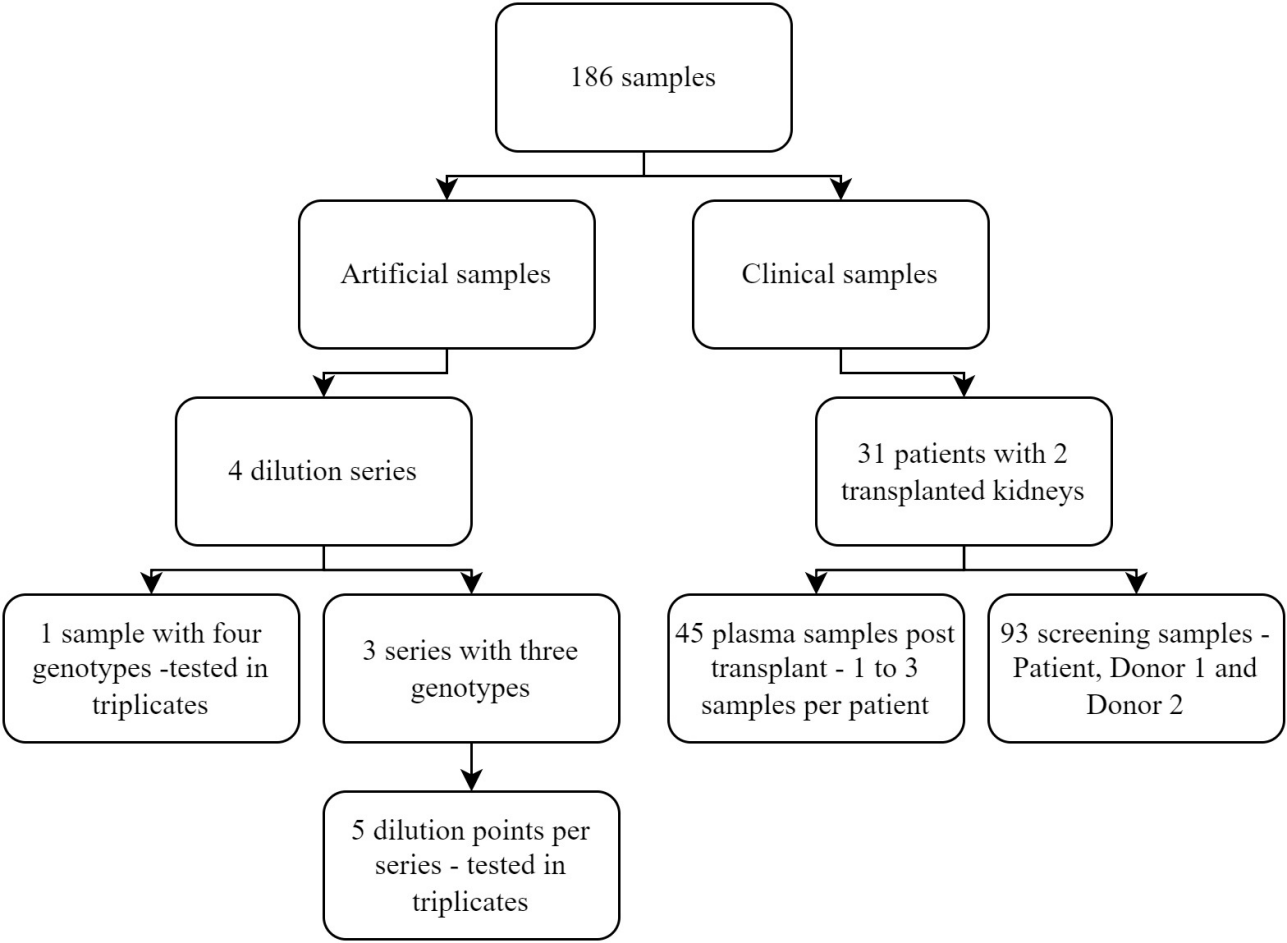
Overview of the different samples included in the study.

A total of 154 clinical and artificial samples were included in the study. The clinical samples originated from 31 patients, who underwent two kidney transplants, and consisted of 93 pre-transplantation DNA samples used for screening and 45 plasma samples used for measuring dd-cfDNA. The artificial samples consisted of 16 samples, including three or four genotypes that were tested in triplicate (**Figure 1**). The artificial samples were prepared with a known percentage of dd-cfDNA for each included donor and used to assess the analytical performance of the assay.

### Artificial samples

To simulate clinical cfDNA samples, eight samples (NA12566, NA20118, NA12476, NA11517, NA12565, NA12348, NA20532, and HG00267) from the Coriell Institute for Medical Research (Coriell) were fragmented by sonication using Bioruptor Pico, 30 s on/off for 25 cycles, to have an average size of 166 bp ( ± 20%). The size of the sonicated samples was analyzed with respect to integrity, purity, and concentration using Agilent TapeStation Cell-free DNA ScreenTape (Agilent Technologies, CA, USA) according to the manufacturer’s instructions. The fragmented Coriell samples were used to simulate patient and donor post-transplant samples. Each sample was diluted to 0.6 ng/µL with 0.1× TE. The DNA concentration was measured using Qubit High Sensitivity Assay (Invitrogen, MA, USA). Samples containing three genotypes were mixed to represent five different genotype ratios, and each sample was tested in triplicate. The sample containing four genotypes was mixed to represent one genotype ratio and tested in triplicate (**Table 1**).

**Table 1:** Theoretical genotype ratios of the artificial samples tested.

	Genotype 1 (%)	Genotype 2 (%)	Genotype 3 (%)	Genotype 4 (%)
**Dilution 1**	94	1	5	–
**Dilution 2**	98.5	0.5	1	–
**Dilution 3**	99.4	0.1	0.5	–
**Dilution 4**	98.9	0.1	1	–
**Dilution 5**	99.8	0.1	0.1	–
**Dilution 6**	98.5	0.5	0.5	0.5

-, Not added.

### Clinical samples

Clinical post-transplantation samples were collected between 2020 and 2022 from 31 patients who had undergone two sequential kidney transplantations and were followed post-transplant at the University Hospital Zurich. The patients gave written informed consent, and the local Cantonal Ethics Committee in Zurich (BASEC-Nr.2018-01182) approved the study. The patients included provided one to three samples each, for a total of 45 samples. In 14 of the 31 patients, the first kidney had been explanted post-transplantation in the setting of nonfunction, but the first kidney remained in 17 of the 31 patients. EDTA-plasma (0.5–1.5 mL) was isolated directly after sampling and stored at −18°C until cfDNA extraction. Frozen plasma samples were thawed and centrifuged for 10 min at 7,830 rpm. The centrifuged plasma samples were then transferred into new K2 EDTA tubes (BD Vacutainer) suitable for the Qiagen QIAsymphony instrument, and 1× PBS was added to a final volume of 2.5 mL for each sample. The cfDNA was extracted using the QIAsymphony DSP Circulating DNA Kit (Qiagen, Hilden, Germany) according to the manufacturer’s instructions. The elution volume for all samples was 45 µL. All cfDNA samples were analyzed with respect to integrity, cfDNA purity, and concentration using Agilent TapeStation Cell-free DNA ScreenTape. The genomic DNA from patients and donors was extracted using an automated device from Maxwell and diluted to approximately 1 ng/µL with 0.1× TE.

### dd-cfDNA analysis with NGS

The One Lambda Devyser Accept cfDNA kit is based on targeted sequencing of 50 indel markers measuring their allele frequency, including a design for the housekeeping gene GAPDH. Each sample was amplified using a single multiplex PCR reaction containing 51 primer pairs to create a target amplicon library (PCR1). The target amplicon library was diluted and used as template in the second PCR (PCR2) where the adapters and unique indices were introduced, enabling pooling of samples into a sequencing run. The indexed libraries were pooled and cleaned to remove buffers, enzymes, and primers using Devyser Library Clean according to the manufacturer’s instructions (Devyser, Stockholm, Sweden) and sequenced in 2 × 75 cycles using either Illumina MiSeq with v3 chemistry or MiniSeq Instruments (Illumina, CA, USA). The turnaround time for this dd-cfDNA assay is typically around 2 days.

The indel amplicons have an average size of 72 bp (including target-specific primers), and the maximum size of the indel amplicons (with insertion and target-specific primers) is 77 bp, enabling almost full coverage with the 2 × 75 cycles sequencing. One primer pair is designed to amplify the housekeeping gene GAPDH with an amplicon size of 321 bp compared to the cfDNA average size of 166 bp. The resulting GAPDH amplicon is included as a system control, and its size compared to cfDNA is suitable for assessing the presence of genomic DNA in the extracted cfDNA sample.

### Relative determination of %dd-cfDNA

To determine the dd-cfDNA, pre-transplant samples were genotyped using the One Lambda Devyser Accept cfDNA assay to identify informative markers. The accompanying software (Advyser Solid Organs) determines the marker genotypes as homozygote (i.e., +/+ or −/−) if the variant allele frequency (VAF) is between 0% and 2%/between 98% and 100% and heterozygote (+/−) if the VAF is between 40% and 60%. If the calculated VAF values are outside these ranges, they are defined as undetermined and the marker will not be used in dd-cfDNA calculations. The software identifies the informative markers for each genotype. A marker was categorized as informative if determined unique for each genotype and thus allowed the measurement of donor DNA in a post-transplant sample. The percentage of donor DNA in a post-transplant sample was determined by using the percentage of donor alleles in the sample for each homozygous informative marker. For each heterozygous informative marker, the percentage of donor alleles found in the sample was determined and multiplied by two. The percentage of the donor in the post-transplant sample was calculated as the average from the results of the informative markers. Informative markers that had a low coverage (<1,000) and outliers in %dd-cfDNA were removed from the average calculations; thus, the SD was below or equal to the %dd-cfDNA.

The Advyser Solid Organs software accommodates two donor profiles, and therefore the artificial samples with three donors were analyzed manually.

### Absolute quantification of dd-cfDNA

Absolute quantification of the dd-cfDNA from each donor was calculated using the concentration and average size determined by TapeStation analysis of the cfDNA sample as well as the volume plasma used for each sample. The percentage dd-cfDNA, determined using One Lambda Devyser Accept cfDNA, was multiplied with the adjusted copy number (cp/mL) [described in Kueng et al. (2023) and Oellerich et al. (2019)] (4, 18).

### Statistical analysis

The open-source programming language R was used for all statistical analyses, and data visualization was performed with RStudio v.2023.06.0 build 421 [R Core Team, 2023 (https://www.r-project.org)] and ggplot2. Wilcoxon rank-sum test was used for statistical testing between cfDNA from the two kidneys. Correlation and regression were calculated using Pearson’s least squares and Passing–Bablok with 95% confidence interval (95% CI, bootstrap, quantile method).

## Results

### Artificial samples

**Figure 2:**
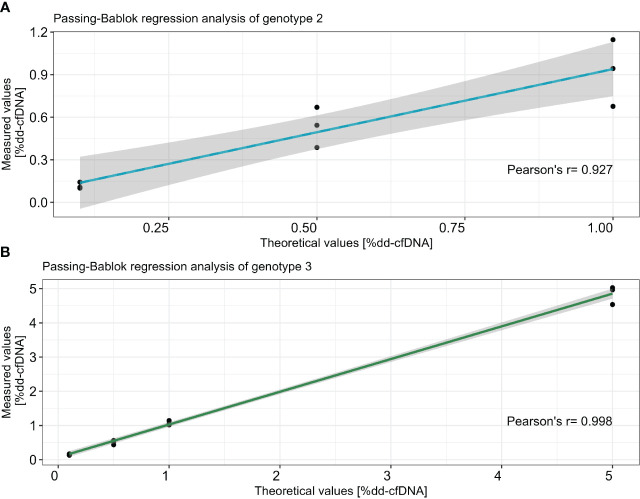
Correlation between theoretical dilution and average of measured %dd-cfDNA in two different dilution series for genotype 2 **(A)** and genotype 3 **(B)**. The *x*-axis represents the theoretical values and the *y*-axis shows the measured values. Passing–Bablok regression with *R* ^2^ is calculated with Pearson’s least squares, and the CI is calculated using the bootstrap method.

Three dilution series containing three genotypes, 15 samples in total, as well as the sample containing four genotypes were tested in triplicate. Each donor had a minimum of three informative markers that were used to determine the %dd-cfDNA (**Table 2**). Importantly, the standard deviation between replicates was below the measured %dd-cfDNA for all dilutions. In the 39 samples where we added 0.1% (LoD_95_) dd-cfDNA from donors 1 and 2, we detected an average of 0.12% dd-cfDNA, with a standard deviation of 0.06 and a coefficient variation (CV) of 49% (data not shown). In the samples where we added 0.5% dd-cfDNA from both donors, we measured a CV between 5% and 25%. The correlation between the theoretical %dd-cfDNA and the measured %dd-cfDNA was high in all three dilution series with two donors. The slope was +1.11 for the second genotype (0.1–1%dd-cfDNA) and 1.00 for the third genotype (0.1–5% dd-cfDNA). The *y*-intercept was 0.01 for the second genotype and 0.03 for the third genotype; the *R* ^2^ value was 0.927 and 0.998, respectively (**Figures 2A, B**). These data display a linear and accurate performance of the assay in the setting of detecting and accurately quantifying dd-cfDNA from artificial samples with two donors with minimal systematic bias.

**Table 2:** Number of informative markers in the artificial dilution series.

Series	Genotype 1	Genotype 2	Genotype 3	Genotype 4
**1**	8	4	8	–
**2**	7	3	5	–
**3**	7	6	8	–
**4**	1	1	3	3

-, Not added.

### Clinical samples

One cfDNA patient sample was excluded due to the low number of sequencing reads as a result of the low amount of available DNA (patient 17). The average DNA input for the remaining 44 that included clinical cfDNA samples was 4.3 ng (ranging from 0.5 to 30.2 ng), and the average cfDNA purity was 82% (ranging from 56% to 95%). The average size of the cfDNA was 236 bp (ranging from 203 to 315 bp). The average read depth for each informative marker was 13,227 read pairs (ranging from 1,033 to 142,394 read pairs), excluding GAPDH.

### Informative markers

Informative markers were detected in 30 out of 31 screened patients for both donor 1 (first transplant) and donor 2 (second transplant). This finding underscores our capability to accurately determine the origin of dd-cfDNA in 97% of cases within our cohort of 31 recipients who underwent two sequential kidney transplants. In the patient without informative markers (patient 29), donor 1 had 11 informative markers and donor 2 had 0. If donor 1 was not in consideration, donor 2 and the patient had four informative markers. However, we did not detect any dd-cfDNA in this patient; hence, the results could be included in the analysis. The average informative markers for donors 1 and 2 were 5.3 (1–11 markers) and 6.0 (0–15 markers), respectively (**Table 3**).

**Table 3:** Number of informative markers in the clinical samples.

Case	Informative markers		
	Patient	Donor 1	Donor 2
**1**	2	1	4
**2**	3	8	8
**3**	2	4^a^	7
**4**	0	8	9
**5**	0	10^a^	5^a^
**6**	7	5	2
**7**	6	5	6
**8**	4	5	7
**9**	2	8	5
**10**	3	5^a^	11
**11**	1	5^a^	13
**12**	1	8	4^a^
**13**	5	8	5
**14**	7	2	2
**15**	2	4^a^	11
**16**	1	4	15
**17**	2	2^a^	9
**18**	4	6	8
**19**	7	8	5
**20**	7	8	3
**21**	7	4	5
**22**	0	4^a^	4^a^
**23**	5	2^a^	7
**24**	4	2^a^	4
**25**	6	7	3
**26**	3	7	4^a^
**27**	4	4	7
**28**	3	3	2
**29**	2	11	0^a^
**30**	5	3	7
**31**	8	3	3

a Related to patient.

### Relative determination of %dd-cfDNA

**Figure 3:**
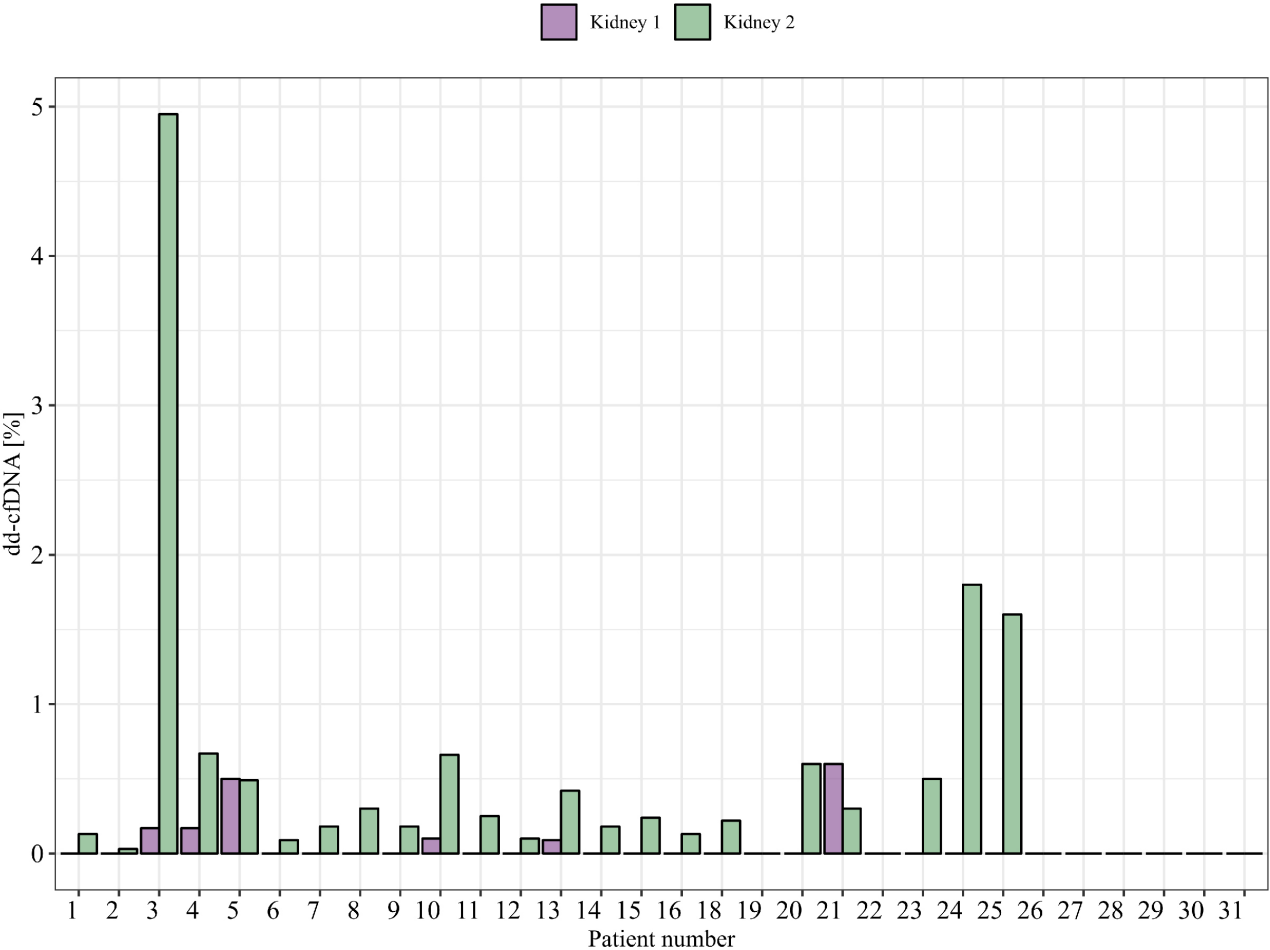
%dd-cfDNA from each donor in clinical samples from patients (one time point is shown).

**Figure 4:**
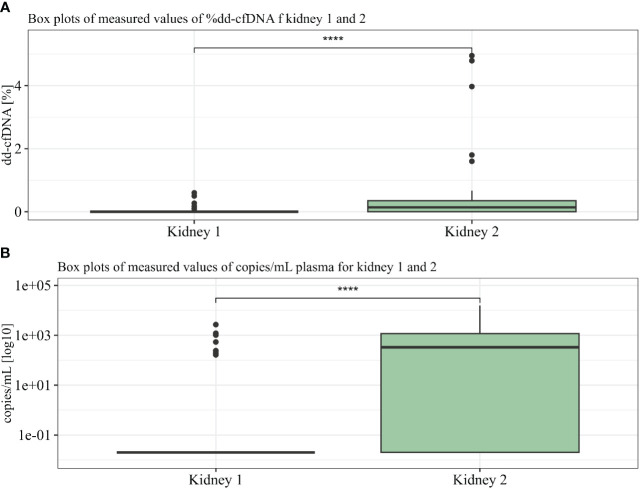
%dd-cfDNA **(A)** and copies cfDNA/mL **(B)** from kidneys 1 and 2 summarized in two boxplots. *U*-test proved significance between differences seen in kidneys 1 and 2. **** p<10^-6^.

The levels of dd-cfDNA above LoD_95_ (>0.1%) from donor 1 were detected in eight (18%) patient cfDNA samples, which represents six of the 30 patients (20%) where we could accurately determine the dd-cfDNA source (**Figure 3**). The average of the detected dd-cfDNA from donor 1 was 0.25% (ranging from 0.1% to 0.6%). The levels of dd-cfDNA from donor 2 were detected in 29 (66%) patient cfDNA samples, with an average of 0.8% (ranging from 0.1% to 4.95%). In all samples where cfDNA from donor 1 was detected, cfDNA from donor 2 was also detected (see **Figure 4**), but %dd-cfDNA and cp/mL from the second kidney were significantly higher on average (Wilcoxon *p* < 0.005) (**Figures 4A, B**).

### Absolute quantification of dd-cfDNA in clinical samples

The relative determination of dd-cfDNA is calculated by using the total amount of cfDNA in the patient. The recipient cfDNA can fluctuate over time due to several processes such as exercise, inflammations, or stress (19). Differences between absolute quantification and relative determination of dd-cfDNA have been seen in late post-transplant patients and can be relevant to assay interpretation (20). Recent studies have also shown a slightly better performance of absolute dd-cfDNA values in predicting molecularly classified ABMR compared to relative values (11).

**Figure 5:**
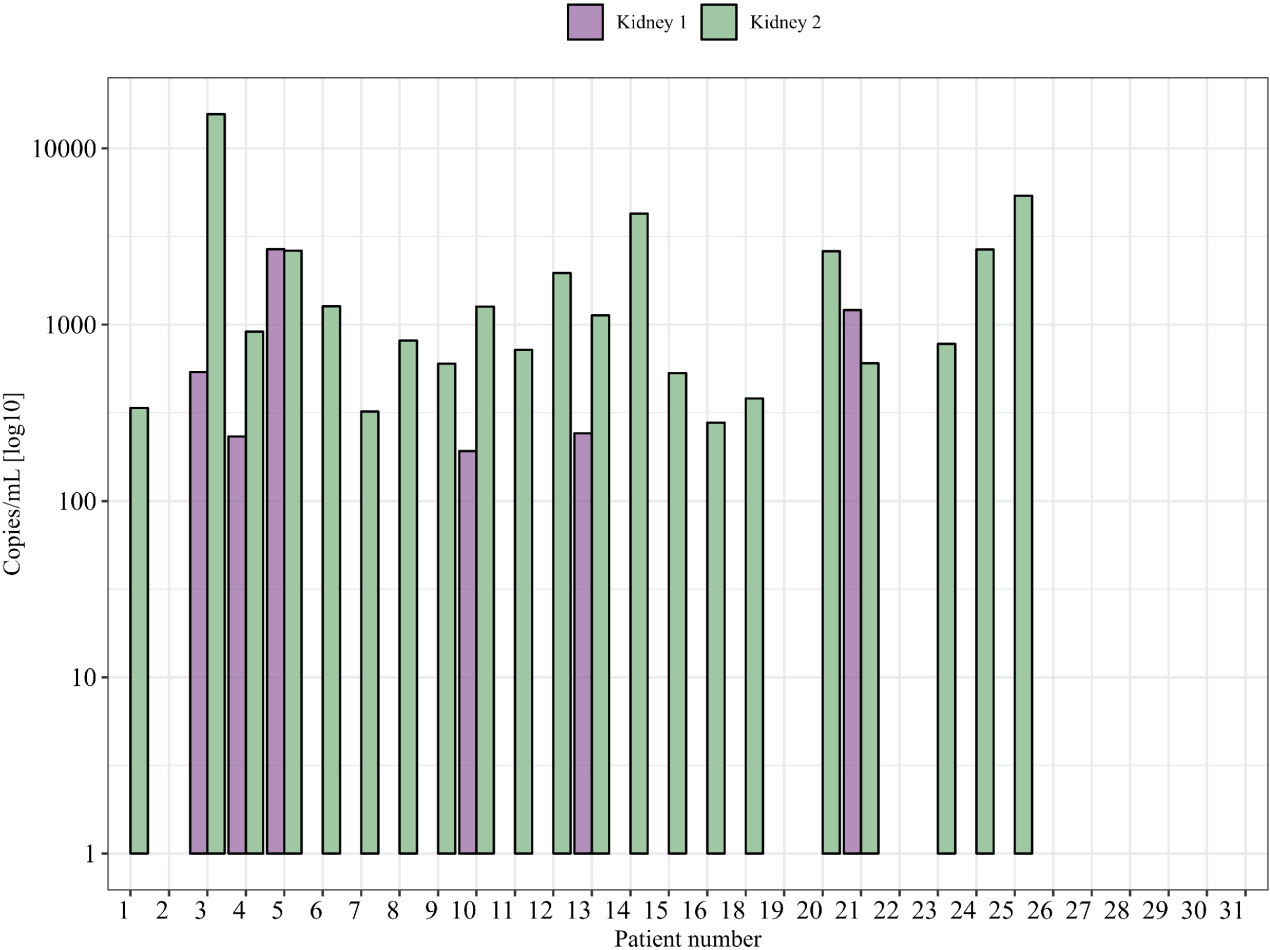
Absolute quantity of dd-cfDNA (cp/mL) from each donor in clinical samples (one time point is shown).

By using the measured size, the concentration for each sample (between 50 and 700 bp), plasma volume, and average amplicon length using One Lambda Devyser Accept cfDNA, the adjusted copy number per milliliter of plasma (cp/mL) was calculated for each sample. The percentage dd-cfDNA was used to calculate the copy number of each donor in the sample. The average copy number for the eight samples containing donor 1 was 784 cp/mL (164–2,681 cp/mL), and for the 29 samples containing donor 2, the average was 4,663 cp/mL (164–15,658 cp/mL) (see **Figure 5**).

### Longitudinal samples and patients with nephrectomies

**Figure 6:**
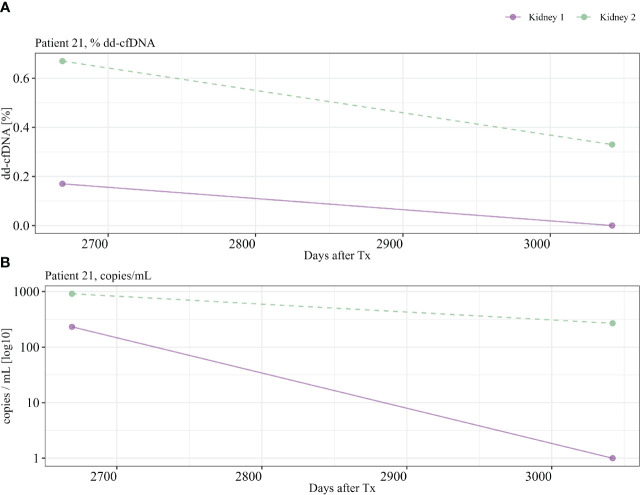
Patient 21 with multiple time points. Detection of dd-cfDNA from kidney 1, >350 days after transplant. **(A)** %dd-cfDNA, **(B)** copies dd-cfDNA/mL.

For patients with samples from multiple time points after transplantation, we could detect some variability over time, in both %dd-cfDNA and copies/mL from donor 1 and donor 2. As an example, patient 21 showed undetectable levels of dd-cfDNA from donor 1 in earlier samples after transplantations that then became positive at a later time point, indicating that the levels of dd-cfDNA can fluctuate also in the setting of a nonfunctioning prior kidney transplant (**Figures 6A, B**). Of note, we were also able to detect dd-cfDNA from donor 1 in one patient (patient 4) where the transplant had been explanted 8 years before the plasma sample was obtained (**Figure 3**). In this sample, we were able to detect elevated values for three of five informative markers, which clearly shows that donor cells able to release dd-cfDNA were still present in the patient. We were, however, not able to detect any dd-cfDNA from the first kidney in 13 other patients included in the study where the first transplant had been explanted, which indicates that this is perhaps an uncommon event (dd-cfDNA detected in one of 14 patients, 7%). For patients where the donor 1 kidney remained, we were able to detect dd-cfDNA from donor 1 in five of 16 patients (31%), showing that a larger amount of remaining graft tissue is, as expected, associated with detectable dd-cfDNA (see **Supplementary Figure S1** for dd-cfDNA in patients with multiple time points).

## Discussion

The post-transplant monitoring of kidney transplant patients is a critical aspect of ensuring graft survival and optimizing patient outcomes. In recent years, the emergence of circulating cfDNA analysis, specifically dd-cfDNA, has provided a promising avenue for noninvasive monitoring. By quantifying the proportion of dd-cfDNA in the recipient’s bloodstream, clinicians can detect early signs of allograft damage and gain valuable insights into graft health.

While dd-cfDNA analysis has potential, it is not without challenges, particularly when monitoring patients who have received two transplanted kidneys. In such cases, the differentiation of the origin of dd-cfDNA from each kidney becomes a complex task. As dd-cfDNA can potentially be shed into the bloodstream by both transplanted kidneys, accurately attributing the source could be important. This would also apply to patients transplanted sequentially with multiple organs from different donors. The monitoring of dd-cfDNA post-transplant could be performed so that values over a certain cutoff will trigger additional invasive graft diagnostics such as biopsies, and accurately determining the source of the shed dd-cfDNA in this setting might therefore assist in reducing the risk associated with these procedures. If dd-cfDNA is being continuously or intermittently released from the first kidney graft, then this might be falsely associated to the second graft, by techniques that are unable to correctly determine the source of the detected dd-cfDNA, and lead to a suspicion of ongoing graft damage.

We present data on a new NGS-based dd-cfDNA assay that is able to quantify dd-cfDNA from multiple grafts both in our *in vitro*-generated mixed samples and in a cohort of patients who have been transplanted with two kidneys. In our opinion, NGS-based dd-cfDNA assays have the ability to combine linear quantification with the ability to analyze multiple samples within a clinically acceptable turnaround time at a cost that allows for multiple analysis. Our data show that the method has a high accuracy and points to the fact that dd-cfDNA from the first kidney can be detected in a clinically relevant subgroup of the analyzed patients (20%, 6/30). Some of the investigated patients with multiple longitudinal samples after transplantation also showed fluctuation in the % detected dd-cfDNA from the first kidney between the samples. This suggests that methods that are unable to accurately distinguish the source of the dd-cfDNA could be subjected to a varying influence from the first graft that could be an additional complication in the ability of using such methods in the post-transplant monitoring of graft function.

In the current study, we were also able to detect small amounts of dd-cfDNA from a graft that had been explanted 8 years before the plasma sample was obtained. Even though this is a single patient and the results should be interpreted with caution, we believe that the result is relevant and points to the fact that donor-derived cells can still remain for a long term after transplantectomy in significant numbers that are able to generate a detectable dd-cfDNA signal. It is tempting to speculate that individuals with a detectable dd-cfDNA signal from the first graft would be at an increased risk of developing DSA against the HLA antigens associated with the first graft. Elevated dd-cfDNA early post-transplantation has also previously been shown to be associated with a high panel reactive antibody (PRA) value, which is a marker of immunological risk (21). Perhaps the monitoring of dd-cfDNA could be used to guide decisions on continued immunosuppressive treatment after transplantectomy in such individuals. An alternative interpretation is that the ongoing release of dd-cfDNA from the remaining cells is a sign of continued immunological destruction and that the remaining graft tissue in individuals without ongoing immunologically mediated tissue destruction would not have detectable dd-cfDNA. If this is the case, the analysis of dd-cfDNA could perhaps not be used to guide immunosuppression as the dd-cfDNA can only be detected after the rejection has already been initiated. The detection of dd-cfDNA in this setting might also be a rare event as we could not detect it in any of the additional 13 patients where the first transplant had also been removed. Further larger longitudinal studies on patients with explanted kidney grafts are needed to pursue these interesting and clinically relevant questions.

The presented assay relies on the use of donor and recipient DNA in an initial one-time screening analysis where these data can then be used to accurately assign the origin of the detected cfDNA. This is an additional step that requires supplementary genetic material. However, most transplant immunological laboratories store donor DNA at the time of transplantation, and if that is not available, additional sources of donor DNA can also be used, such as graft biopsies. The data presented in the current study are also relevant for patients with multiple organ transplants (for example, lung and kidney or liver and kidney) from different donors where accurate donor assignment of dd-cfDNA is also central. Previous studies have also shown much higher normal dd-cfDNA values in the setting of liver and lung transplantation, which might preclude the analysis of kidney-derived dd-cfDNA in such individuals if accurate donor assignment is not accomplished (22, 23).

In summary, we present data on a new NGS-based dd-cfDNA analysis that is able to detect and separate dd-cfDNA from multiple sources both in artificially generated samples and in patients with two kidney transplants. Our data have clinically important implications for the monitoring of dd-cfDNA post-transplantation and suggest that the accurate determination of the origin of dd-cfDNA is both relevant and feasible. Additional larger studies are needed to confirm our findings.

## Data availability statement

The raw data supporting the conclusions of this article will be made available by the authors, without undue reservation.

## Ethics statement

The studies involving humans were approved by the local Cantonal Ethics Committee in Zurich (BASEC-Nr.2018-01182). The studies were conducted in accordance with the local legislation and institutional requirements. The participants provided their written informed consent to participate in this study.

## Author contributions

LP: Formal analysis, Investigation, Writing – original draft, Writing – review & editing, Data curation, Methodology. LF: Formal analysis, Investigation, Methodology, Writing – review & editing. SW: Investigation, Methodology, Writing – review & editing, Data curation, Visualization. HR: Data curation, Methodology, Writing – review & editing, Formal analysis, Software. LW: Data curation, Methodology, Writing – review & editing, Investigation. KC: Data curation, Investigation, Methodology, Writing – review & editing. TS: Data curation, Investigation, Methodology, Writing – review & editing, Formal analysis. JN: Data curation, Formal analysis, Investigation, Methodology, Supervision, Writing – review & editing, Conceptualization, Funding acquisition, Project administration, Writing – original draft.

## Supplementary material

Supplementary Figure 1 Additional patients with multiple time points. %dd-cfDNA **(A)** and copies dd-cfDNA/mL **(B)** is shown for each patient.

Supplementary Figure 2 Chromatogram of cfDNA clinical samples analyzed with TapeStation.
