# Supplementary material for: Detection of donor-derived cell-free DNA in the setting of multiple kidney transplantations
Source: Front Immunol. 2024 Feb 22;15:1282521. doi: 10.3389/fimmu.2024.1282521 (PMC10917974; doi:10.3389/fimmu.2024.1282521)
Supplement: Supplementary file 1 [file DataSheet_1.pdf]

Supplementary Table 1. Clinical data

Clinical data			
**Patient**	**Transplant ectomy 1st graft**	**Reason for graft loss 1st graft**	**Living related donor 1st graft**
**1**	Yes	Thrombosis	No
**2**	Yes	BK-Virus nephritis	No
**3**	No	CNI-toxicity	Yes
**4**	Yes	Thrombosis	No
**5**	No	ABMR	Yes
**6**	Yes	BK-Virus nephritis	No
**7**	No	FSGS-relapse	No
**8**	No	SLE-nephritis relapse	Yes
**9**	No	CNI-toxicity	Yes
**10**	No	FSGS-relapse	Yes
**11**	No	ABMR	Yes
**12**	Yes	Thrombosis	No
**13**	No	Thrombosis	No
**14**	No	ABMR	No
**15**	No	ABMR	No
**16**	No	ABMR	No
**17**	No	ABMR	Yes
**18**	Yes	Thrombosis	No
**19**	Yes	Primary non function	No
**20**	Yes	Thrombosis	No
**21**	No	Primary non function	No
**22**	Yes	ABMR	Yes
**23**	No	ABMR	Yes
**24**	No	ABMR	Yes
**25**	Yes	ABMR	No
**26**	Yes	IgA nephritis relapse	Yes
**27**	No	IgA nephritis relapse	No
**28**	Yes	Thrombosis	No
**29**	No	ABMR	No
**30**	Yes	ABMR	No
**31**	Yes	Thrombosis	No
