# Supplementary figures and images for: Detection of donor-derived cell-free DNA in the setting of multiple kidney transplantations
Source: Front Immunol. 2024 Feb 22;15:1282521. doi: 10.3389/fimmu.2024.1282521 (PMC10917974; doi:10.3389/fimmu.2024.1282521)

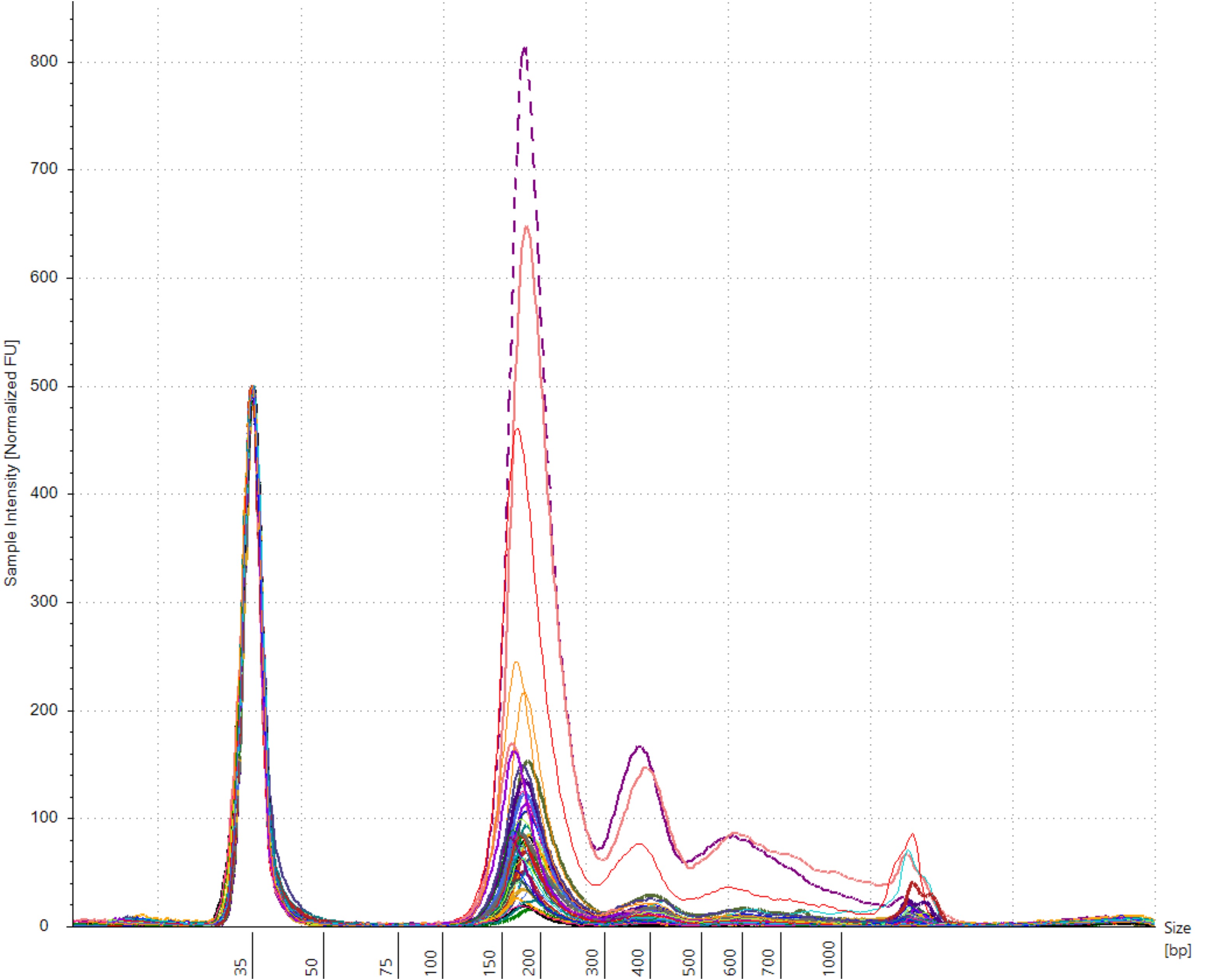

Supplement: Supplementary Figure 1 — Additional patients with multiple time points. %dd-cfDNA (A) and copies dd-cfDNA/mL (B) is shown for each patient. [file Image_1.jpeg]

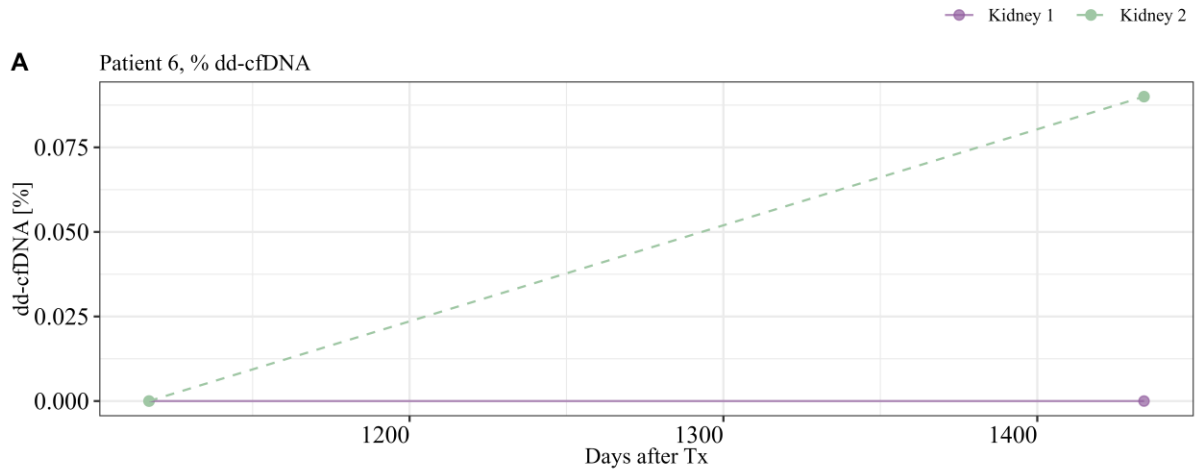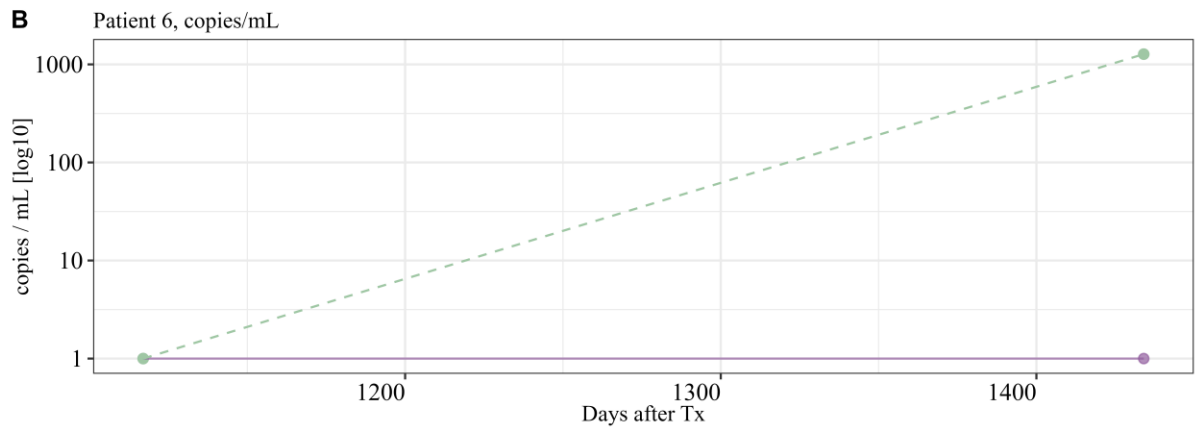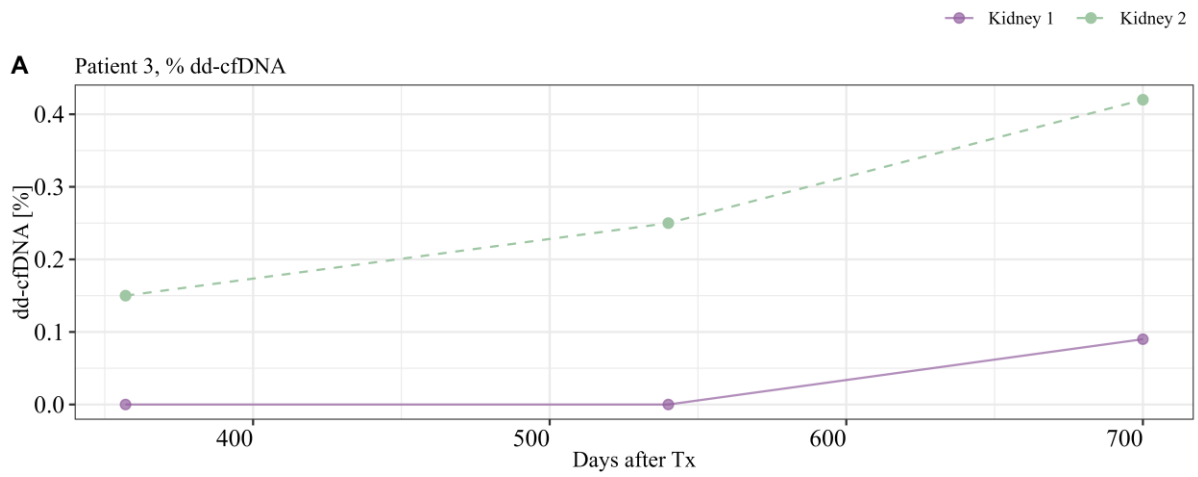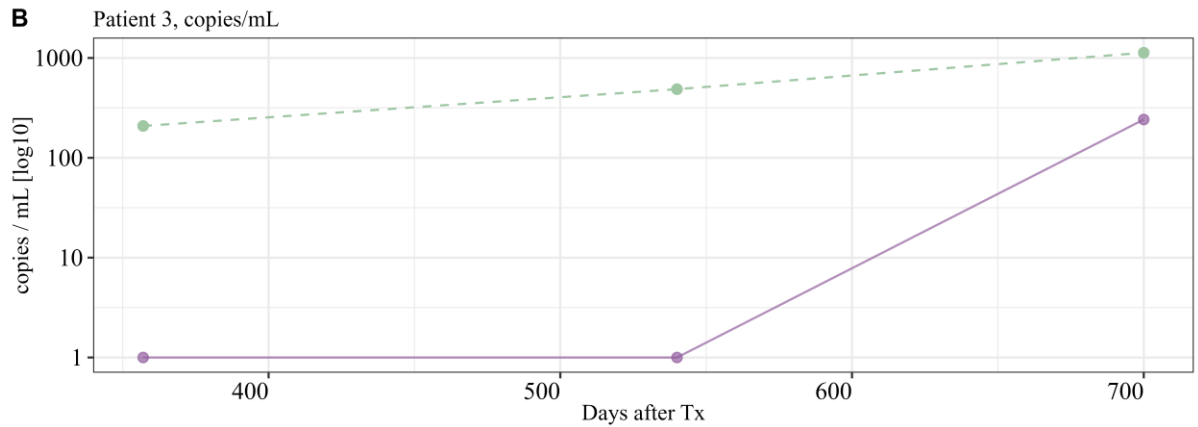

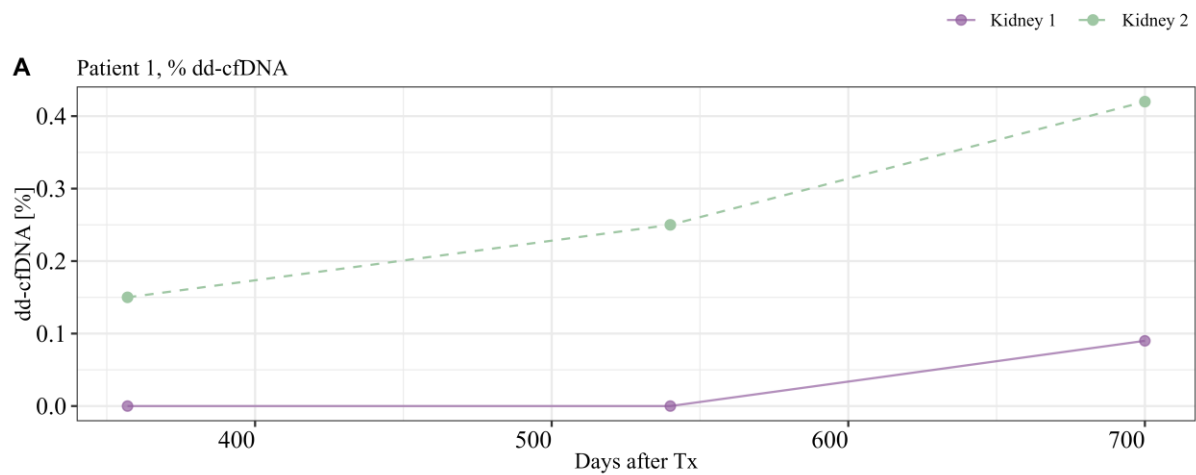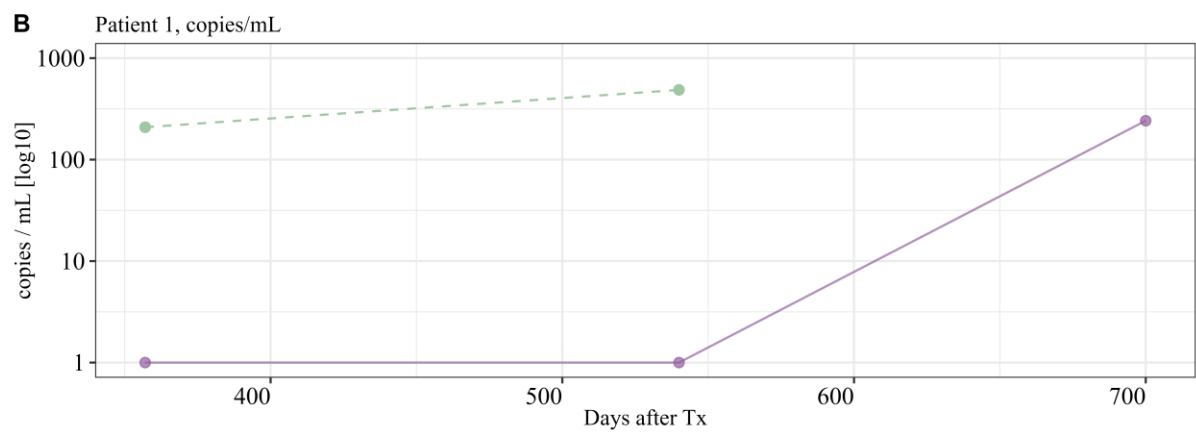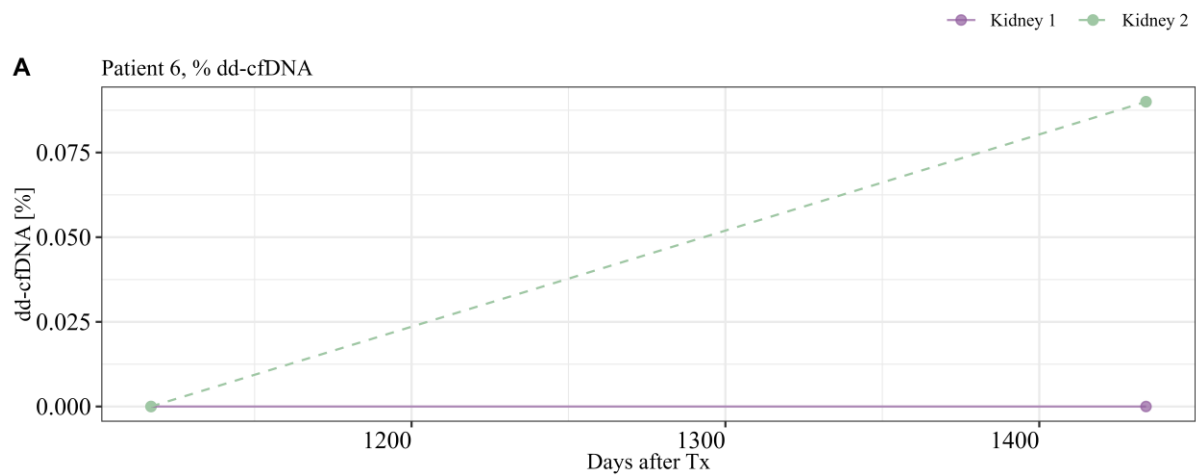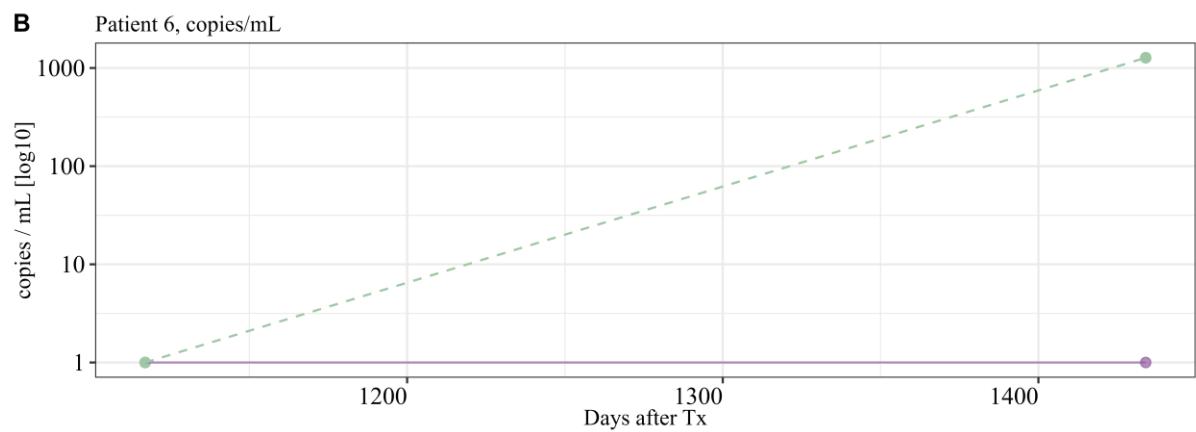

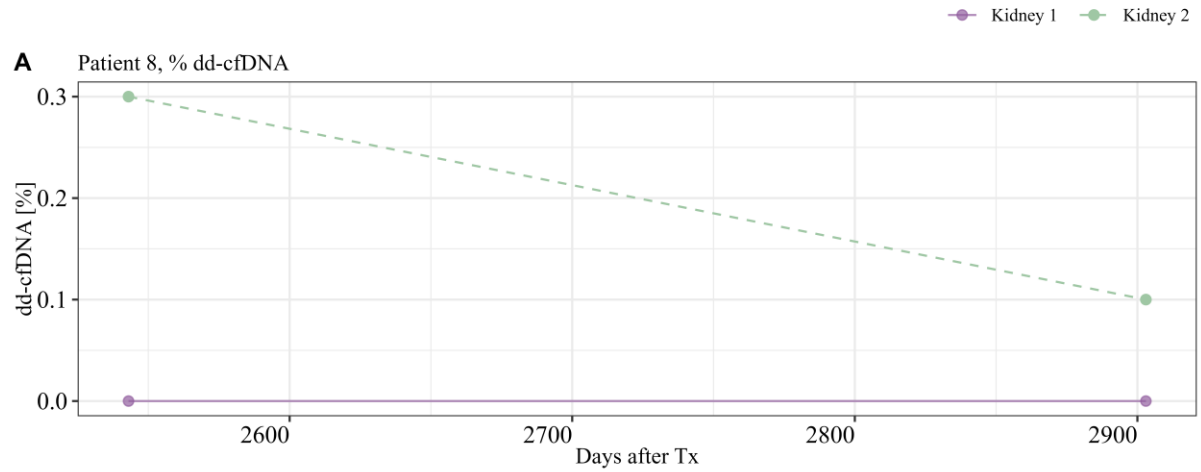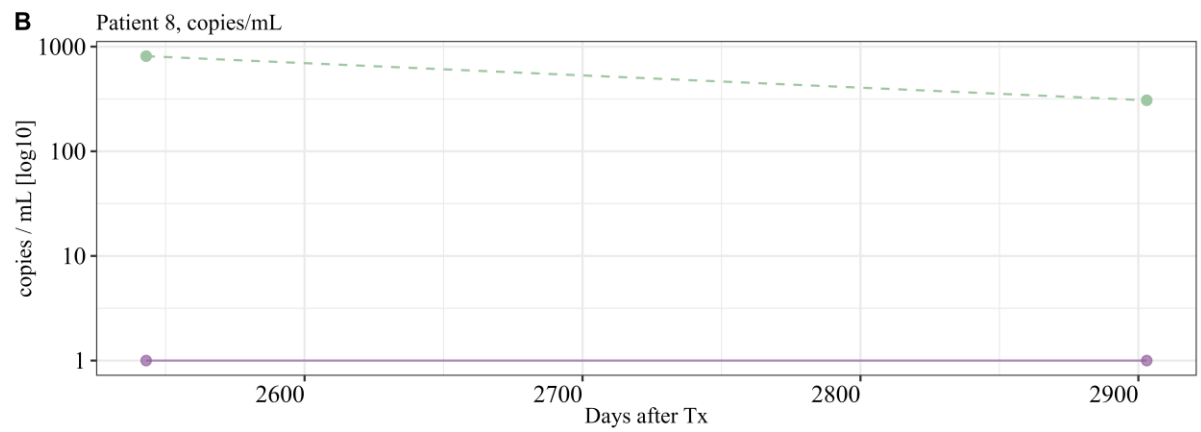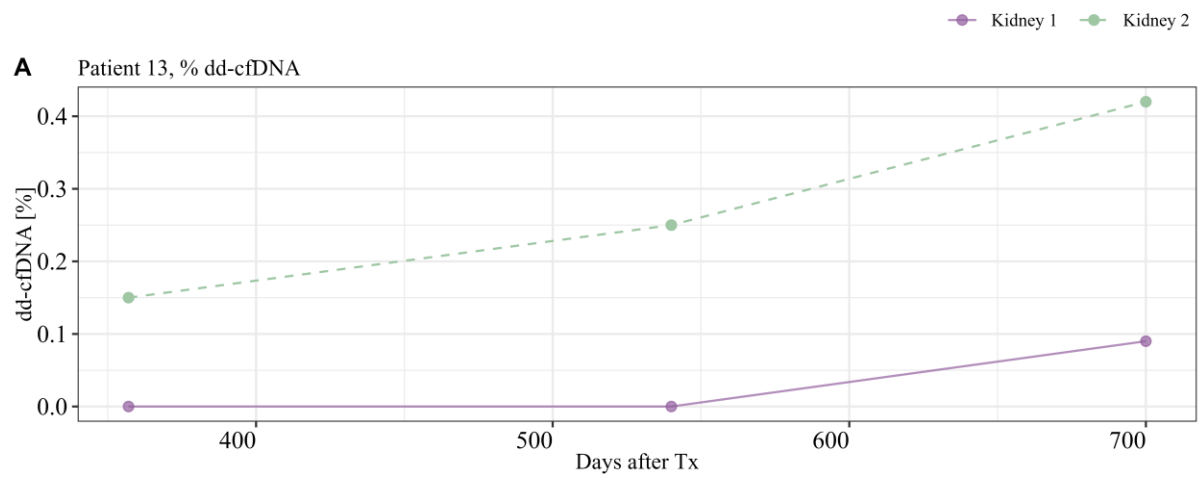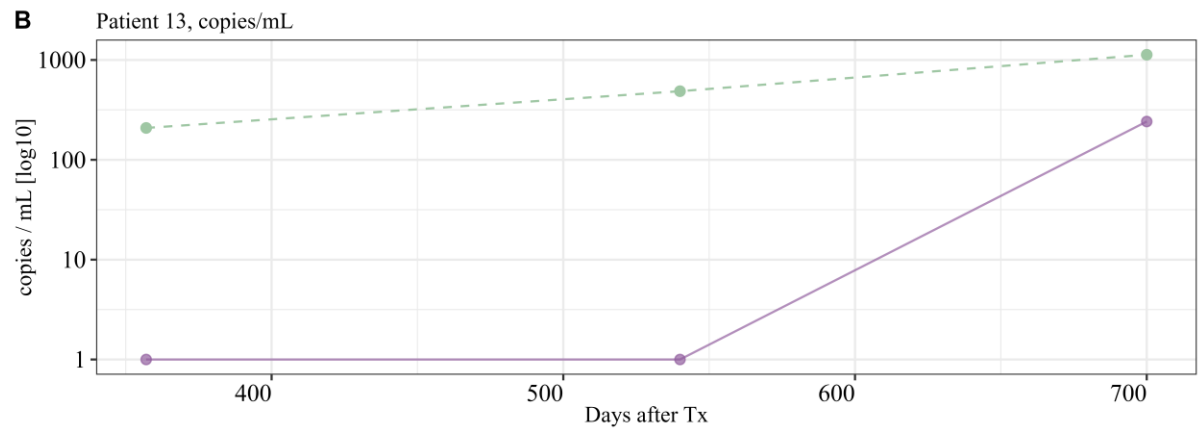

Supplement: Supplementary Figure 2 — Chromatogram of cfDNA clinical samples analyzed with TapeStation. [file DataSheet_2.pdf]
